# Inflammasome Adaptor ASC Is Highly Elevated in Lung Over Plasma and Relates to Inflammation and Lung Diffusion in the Absence of Speck Formation

**DOI:** 10.3389/fimmu.2020.00461

**Published:** 2020-03-19

**Authors:** Mikhail A. Gavrilin, Christian C. McAndrew, Evan R. Prather, MuChun Tsai, Carleen R. Spitzer, Min-Ae Song, Srabani Mitra, Anasuya Sarkar, Peter G. Shields, Philip T. Diaz, Mark D. Wewers

**Affiliations:** ^1^Pulmonary, Critical Care and Sleep Medicine Division, Department of Internal Medicine, The Ohio State University, Columbus, OH, United States; ^2^Davis Heart and Lung Research Institute, Ohio State University, Columbus, OH, United States; ^3^College of Public Health, The Ohio State University, Columbus, OH, United States; ^4^Comprehensive Cancer Center, James Cancer Hospital, The Ohio State University, Columbus, OH, United States

**Keywords:** bronchoalveolar lavage, macrophages, inflammasomes, lung, ASC, smoking, HIV, pneumonia

## Abstract

**Rationale:** Caspase-1 is a zymogen whose activation predominantly depends upon the assembly of ASC monomers into insoluble prion-like polymers (specks). ASC polymers support caspase-1 dimer formation inducing a proximity mediated auto-activation of caspase-1. Therefore, the amount and nature of ASC monomers and polymers in lung bronchoalveolar lavage fluid (BALF) might serve as a marker of lung inflammasome activity.

**Objectives:** To determine whether lung ASC concentrations or oligomerization status predicts lung function or activity of lung inflammation.

**Methods:** BALF ASC amount and oligomerization status was studied in three distinct cohorts: (1) young healthy non-smokers, vapers and smokers; (2) healthy HIV+ smokers who underwent detailed lung function studies; and (3) hospitalized patients with suspected pneumonia. We quantified cell free BALF ASC levels by ELISA and immunoblot. Oligomers (i.e., ASC specks) were identified by chemical crosslinking and ability to sediment with centrifugation.

**Measurement and Main Results:** ASC levels are significantly higher in lung lining fluid than in plasma as well as higher in smoker lungs compared to non-smoker lungs. In this context, ASC levels correlate with macrophage numbers, smoking intensity and loss of lung diffusion capacity in a well-characterized cohort of healthy HIV+ smokers. However, only monomeric ASC was found in our BALF samples from all subjects, including patients with lung infections.

**Conclusions:** Even though, most, if not all, extracellular ASC in BALF exists in the soluble, monomeric form, monomeric ASC concentrations still reflect the inflammatory status of the lung microenvironment and correlate with loss of lung function.

## Introduction

ASC (PYCARD) is a 22 kDa protein first discovered as a caspase recruitment domain (CARD)-containing protein that aggregates into insoluble specks during mononuclear phagocyte apoptosis (1–3). ASC's first protein partnership was found to be with pyrin (the protein responsible for familial Mediterranean fever) (4, 5) via pyrin domain (PYD) interactions (6). Shortly thereafter ASC was found to link caspase-1 and PYD-containing NOD-like receptor sensors (NLRs) into inflammasomes, protein complexes that promote caspase-1 activation (7). It is currently accepted that ASC polymers provide a platform to bring procaspase-1 molecules into close proximity, mediating their auto-activation (8). Specifically, polymeric ASC's CARD domains are thought to cause homo-dimerization of caspase-1 CARDs thus inducing auto-activation by the enzymatic removal of a p10 fragment from the p45 procaspase-1. The resulting p35 caspase-1 requires its CARD mediated association with the ASC polymer to maintain its catalytic function (9). Thus, ASC polymers (i.e., specks) are often used as reliable indicators of inflammasome assembly (9, 10).

Cytosolic ASC is released from mononuclear phagocytes after activation of the inflammasome in both monomeric and oligomeric forms along with cleaved caspase-1, IL-1β, and IL-18 (10–12). This released ASC can be visualized as specks that are often found associated with the producer cell but may also be released into the extracellular milieu where these specks have been reported to have prion-like activities that promote inflammation (11, 13, 14). For example, Franklin et al. (11) measured ASC in BALF samples and suggested that this ASC exists as specks that may be responsible in part for the chronic inflammation seen in COPD patients. We therefore determined to test this hypothesis in several cohorts of subjects to determine whether BAL ASC exists in a prion-like speck form or not; and whether the absolute ASC concentration and oligomerization status of the BAL ASC relates to lung function in smokers or active inflammatory conditions.

## Materials and Methods

### ELISA

The ASC ELISA was developed in our laboratory using a sandwich design with capture monoclonal ASC antibody [Anti-ASC (TMS1), clone 23-4, MBL International, Woburn, MA], a rabbit polyclonal detection antibody developed in our laboratory (15), and a secondary goat anti-rabbit polyclonal antibody conjugated to horse-radish peroxidase (HRP). The human ASC standard was recombinant MBP-ASC purified on xylose beads. ASC purity was determined by Coomassie blue staining and concentration determined by Lowry protein assay and absorption at A_280_ using E1% = 0.951 predicted from the ExPASy ProtParam tool for the MBP-ASC hybrid (Swiss Institute of Bioinformatics).

### ASC Immunoblot

Total protein estimations were done by Lowry assay (BIO-RAD, Hercules, CA) where mentioned. Samples were diluted in Laemmli sample buffer (BIO-RAD, Hercules, CA) containing 10% 2-mercaptoethanol (Millipore Sigma, St. Louis, MO), and protein separation was performed by sodium dodecyl sulfide-polyacrylamide electrophoresis (SDS-PAGE) using a 4-12% bis-Tris gel. Proteins were transferred to a polyvinylidene difluoride (PVDF) membrane and then blocked in 5% milk in Tris-buffered saline containing 1% Tween-20. The membrane was incubated with an anti-ASC rabbit polyclonal antibody developed in our laboratory (15), washed, and then incubated with a donkey anti-rabbit secondary antibody conjugated to HRP (GE Healthcare, UK). Antibody-bound protein was visualized by autoradiography using enhanced chemiluminescence (ECL, Pierce Thermo Scientific) on film (HyBlot CL, Denville Scientific, Hollston, MA).

### Specificity of ASC Immunoblot Signal

To determine if high molecular weight signals from ASC crosslinking were specific, we pretreated the rabbit ASC antiserum diluted 1:5,000 in milk for 4 h at 4°C with either 7 μg MBP-ASC or nothing. Parallel gels containing identical ASC samples were run as described above and then immunoblotted using either the control or ASC pre-blocked antisera. The parallel immunoblots were compared using a blink comparator animation to identify which bands were specific for ASC.

### Generation of THP-1 Cells Stably Expressing YFP-ASC

Lentiviruses coding YFP-ASC fusion protein were produced as we described earlier in detail (16). THP-1 cells (ATCC, lot 385653, Manassas, VA) were transduced with 2 multiplicities of infection (MOI) of lentivirus by spinoculation in 12-well plates at 1,600 rpm for 1 h in a benchtop Beckman centrifuge. To enhance the efficiency of infection, 0.8 μg/ml of polybrene (Sigma-Aldrich) was used. Immediately following the spinoculation, cells were replenished with fresh culture media and left to recover in a CO2 incubator. Using fluorescent microscopy, we estimated that efficiency of transduction is over 50%. Stably transduced cells were sorted with FACS ARIA III (BD Biosciences) resulting in nearly 100% fluorescent cells. After sorting, cells were cultured in RPMI 1640 conditioned media supplemented with 20% FBS and 1% penicillin\streptomycin (Gibco Laboratories, Gaithersburg, MD) and validated as we described earlier (17).

### Cell Culture of THP-1

THP-1 cells (ATCC, lot 385653, Manassas, VA) were cultured in RPMI media (Corning Inc., Corning, NY) containing 10% fetal bovine serum (Atlas Biologicals, Fort Collins, CO) and 1% penicillin\streptomycin (Gibco Laboratories, Gaithersburg, MD). Cells were screened for mycoplasma by PCR as described (18).

### Processing of Bronchoalveolar Lavage Fluid

IRB-approved BALF samples were collected from three donor cohorts: normal healthy volunteers; HIV1+ healthy volunteers (see HIV cohort description below) and hospitalized patients with suspected pneumonia. For the healthy subjects, BAL was performed with 5 sequential 20 ml saline aliquots into the right middle lobe or lingula (IRB 2005H0197 and IRB 2015C008). For samples obtained from patients with suspected pneumonia, BAL was performed in the presumed infection site (IRB2016H0009). The BALF was spun at 500 g for 5 min to pellet cells, and the supernatant was collected, aliquoted and stored at −80°C until assayed. Cell counts were done by hemocytometer and cell differential counts by staining of Cytospin (Cytospin 3, Shandon, Cambridge Scientific, Watertown, MA) prepared fresh samples using DiffQuik [HEMA3, Fisher, Kalamazoo, MI of as previously described (19).

### Study Populations

#### Healthy Subject Cohort

Forty-three healthy young volunteers were studied from a cohort that included non-smokers (*n* = 12), active smokers (*n* = 16) and exclusive e-cigarette users (*n* = 15) who underwent BAL (IRB 2015C008) as previously reported (20).

#### HIV Smoker Cohort

We included 74 HIV-positive subjects previously enrolled in a prospective study analyzing the effects of smoking on lung innate host responses by BAL (IRB 2005H0197). The subject characteristics are summarized in Table 1. All participants were smokers with an average of 26.2 ± 23.6 pack-years, saliva cotinine concentrations averaged 220 ± 176 ng/ml; 82% were males, and 46% had detectable viral loads. Pulmonary function tests and research bronchoscopies with BAL samples were also obtained for all participants. BAL samples were centrifuged at 500 g × 5 min and the supernatants frozen at −80°C.

**Table 1:** Characteristics of HIV+ smokers in Cohort 2^*^.

Age, yr	42.9 ± 1.1
Sex (M/F)	61/13
BMI	27.5 ± 0.6
Pack-years	26.2 ± 2.6
Race (white/other)	46/28
Detectable viral load (Y/N)	34/40
Viral load (if detectable)	79,452 ± 30,083
Saliva cotinine (ng/ml)	220 ± 21

* *Continuous variables show means ± SEM*.

#### Pneumonia Cohort

Forty hospitalized patients (see Table 3 for demographics) who underwent clinically indicated BAL for suspected pneumonia consented to have residual BALF analyzed for innate immune molecules in the fluid as part of IRB016H0009.

### Chemical Crosslinking of ASC Oligomers

Chemical crosslinking of ASC oligomers was achieved by incubation of BALF or THP-1 lysate in 2 mM disuccinimidyl suberate (DSS) (Pierce) for 30 min at room temperature. The reaction was then quenched by adding Laemmli sample buffer containing 10% 2-mercaptoethanol and samples boiled before SDS-PAGE. For ELISA measurements, the DSS was quenched by adding Tris buffer.

### *In vitro* Assembly of ASC Specks

*In vitro* assembly of ASC specks was performed according to the protocol previously described (21). Briefly, THP-1 cells were washed with phosphate buffered saline (PBS), pelleted at 500 g × 5 min and pellets stored at −80°C in aliquots of 30 × 10^6^ cells until use. Cells were lysed in 100 μl of CHAPS buffer (20 mM HEPES-KOH, pH 7.4, 5 mM MgCl_2_, 0.5 mM EGTA, 0.1% CHAPS) supplemented with phenylmethylsulfonyl fluoride (PMSF) and protease inhibitor cocktail (containing aprotinin, bestatin, E-64, leupeptin and pepstatin A, Sigma Aldrich, St. Louis, MO) by 3 × 20 slow strokes of a 22.5 gauge needle with syringe on ice (intervals done to avoid heating) and cell debris removed by centrifugation at 16,000 g for 10 min at 4°C. Cell extract supernatant was transferred to another pre-chilled tube, and one 40 μl aliquot was incubated for 40 min at 37°C (to induce specks) while other 40 μl aliquot was kept on ice as a speck-free control. At the end of incubation, 360 μl of PBS was added to each 40 μl of cell extract supernatant. In the case of YFP-ASC cells, fluorescent YFP-ASC specks were detected by fluorescent microscopy. To confirm ASC oligomerization by immunoblot, we performed ASC crosslinking with 1–2 mM DSS for 30 min at room temperature.

### Statistical Analysis

Statistical analysis was performed using JMP 14.0 (SAS Institute, Cary, NC). ELISA measurements between all patient groups were expressed as median and the interquartile range. Non-parametric pair wise comparisons used the Wilcoxon method and for multiple comparisons the Steel-Dwass method with *p* < 0.05 accepted as statistically significant. Correlations between continuous variables used Pearson's correlation coefficient.

## Results

### Extracellular ASC Is Highly Concentrated in the Epithelial Lining Fluid

**Figure 1:**
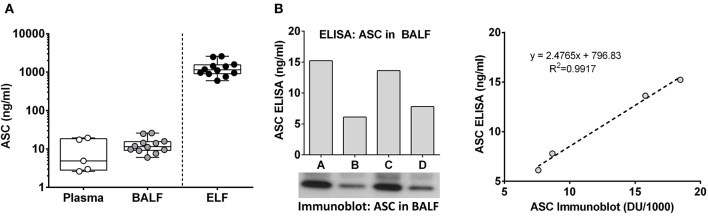
Extracellular ASC is highly concentrated in lung epithelial lining fluid. ASC concentration in plasma and BALF from normal human volunteers was determined by ELISA. Epithelial lining fluid (ELF) concentrations were inferred from the approximate 100-fold dilution that is expected from the BAL procedure, **(A)** ASC values from ELISA (ng/ml) and immunoblot of four random donors are shown along with the Pearson's correlation plotting ng/ml vs. densitometric units **(B)**.

To better understand the relevance of lung airway ASC and its effects on lung physiology, we compared plasma ASC levels in healthy non-smokers to ASC levels in BALF in a healthy cohort of non-smokers. To our surprise BALF ASC, which is about 100-fold diluted from epithelial lining fluid (22), has a similar ASC level compared to plasma ASC 11.4 [9.1–15.4] ng/ml vs. 4.9 [2.8–19.4] ng/ml [median [IQR]], respectively (Figure 1A). Immunoblots of random BALF samples confirmed the specificity of the ASC ELISA measurements (Figure 1B).

To eliminate the possibility that saline used for the BAL induced ASC release after cell harvesting, we compared ASC release in cultured human THP-1 cells in the presence of saline vs. media. Incubation in saline had no noticeable effect on ASC release from mononuclear phagocytes (Supplementary Figure 1). Taken together these findings suggest that BALF ASC levels reflect *in vivo* concentrations, not release postharvest.

### BALF ASC Levels Reflect Lung Inflammation

**Figure 2:**
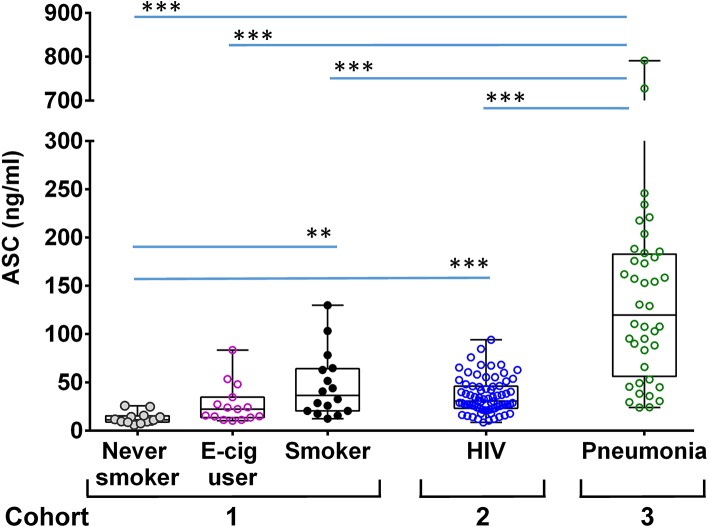
ASC concentration in BALF of different groups of donors. Shown are ASC measures in unconcentrated cell free BALF measured by sandwich ELISA from the following cohorts: a group of never smokers, *n* = 12, e-cigarette users, *n* = 16 and smokers, *n* = 15 [as previously reported (20)]; an HIV smoker cohort, *n* = 74; and hospitalized patients with suspected pneumonia, *n* = 40. Shown are the median and quantiles; ***p < 0.001 and **p < 0.002 by non-parametric comparisons for all pairs using Steel-Dwass method.

To better understand the role of ASC in lung physiology we analyzed a cross-section of individuals with varying baseline exposure conditions. We compared bronchoalveolar lavage fluid (BALF) ASC levels between three cohorts: a cohort of normal young volunteers (divided into non-smoking, e-cigarette smoking and smoking groups) that we had previously reported on (20), a cohort of stable HIV infected smokers who underwent detailed pulmonary function studies, and a cohort of hospitalized patients with suspected pneumonia (Figure 2). As shown, ASC levels were higher in normal smokers [median 36.7 [interquartile range 20.5–64.2] ng/ml] and HIV infected smokers [30.9 [25.3–46.2] ng/ml] than in non-smokers [11.4 [9.1–15.4] ng/ml], *p* < 0.001 and *p* < 0.0001, respectively. However, patients with suspected pneumonia had the highest levels overall 119.9 [56.2–182.9] ng/ml, *p* < 0.0001 for all comparisons.

### Lung ASC Levels Correlate With Smoking Intensity and Lung Diffusion Capacity

**Figure 3:**
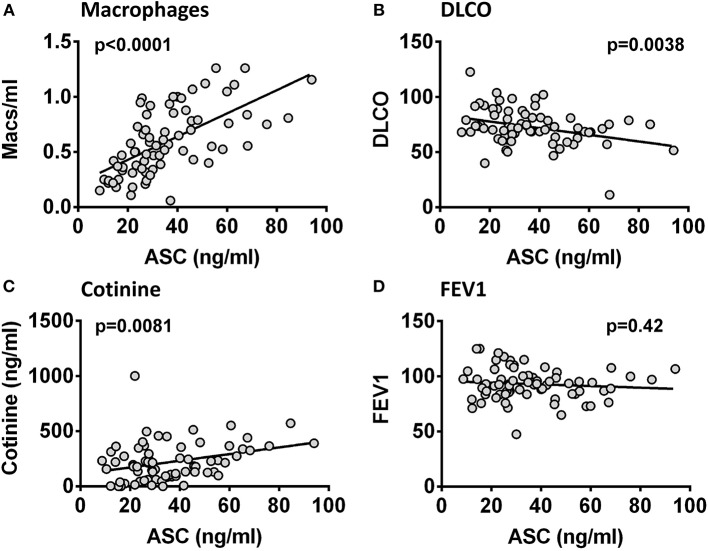
ASC in BALF correlates with macrophage numbers, smoking intensity and lung diffusing capacity. ASC in 74 HIV+ smoker BALF samples compared to macrophage counts (cell number/ml) **(A)**, saliva cotinine (ng/ml) **(B)**, DLCO (% predicted) **(C)**, and FEV1 (% predicted) **(D)**. Correlations were significant for macrophage number (*p* < 0.0001), for cotinine (*p* = 0.0081), and for DLCO (*p* = 0.0038) but not for FEV1 (*p* = 0.42) by Pearson's coefficient.

ASC is released by mononuclear phagocytes upon inflammasome activation (23) and this release has been linked to emphysema (11, 24). Therefore, to assess the clinical significance of the ASC concentrations in BALF we analyzed our HIV+ smoking cohort from a prospective study that studied the relationships between smoking and lung physiology. This cohort represented a generally healthy smoking population of predominantly male HIV+ individuals with low or undetectable viral loads and a significant active smoking history as confirmed by saliva cotinines (a measure of smoking intensity) (Table 1). We compared BALF ASC levels to lung cell yields, smoking intensity and lung function as measured by participants' performance on formal pulmonary function tests. Pulmonary function measurements averaged in the normal range except for lung diffusion capacity (DLCO) (Table 2), which was decreased. This was consistent with previous studies (25, 26). As shown in Figure 3, BALF ASC levels correlated with the concentration of alveolar macrophages in BALF (*p* < 0.0001), with saliva cotinines (*p* = 0.0081), and, most notably, with loss of lung diffusion capacity (*p* = 0.0038). FEV1, total lung capacity and residual volume did not correlate with BALF ASC levels (Supplementary Table 1). Although detectable HIV viral copies were found in the blood of 34 of the 74 subjects (median 4699, IQR 58,873–213, copies/ml), active viral detection had no statistical relationship with BALF ASC levels or pulmonary function testing. Thus, lung ASC levels correlate with loss of lung diffusion capacity and with the degree of macrophage alveolitis.

**Table 2:** Lung function characteristics of subjects in Cohort 2.

Lung function^*^	
FEV1 (L)	3.32 ± 0.80 (89%)
FVC (L)	4.57 ± 1.08 (93%)
FEV1/FVC %	73.1 ± 10.6
FEV1 (post BD) (L)	3.49 ± 0.83 (93%)
FVC (post BD) (L)	4.63 ± 1.04 (99%)
FEV1/FVC % (post BD)	75.3 ± 8.2
TLC (L)	6.64 ± 1.13 (100%)
RV (L)	2.06 ± 0.59 (115%)
DLCO	23.4 ± 5.6 (73%)

* *Spirometric values include pre and post bronchodilator challenge (post BD), lung volumes total lung capacity (TLC), and residual volume (RV) done by body box plethysmography and lung diffusion capacity (DLCO) done by single breath carbon monoxide expressed as mean ± SEM. Parentheses show percent predicted based upon height and age*.

**Table 3:** Characteristics of pneumonia Cohort 3^*^.

Age	55.2 ± 2.4
Sex (M/F)	21/19
Race (white/other)	33/7
On ventilator	22/14
BAL culture (pos/neg)	17/22
Final diagnosis	
Pneumonia	26**
Other	14
PMN% in BALF	50 ± 5.6
Macrophage% in BALF	42.2 ± 5.8

* Continuous variables show means ± SEM. **“pneumonia” defined by consult team's final assessment, whereas “other” denotes final diagnosis uncertain.

### Speck Formation Affects ASC Detection

**Figure 4:**
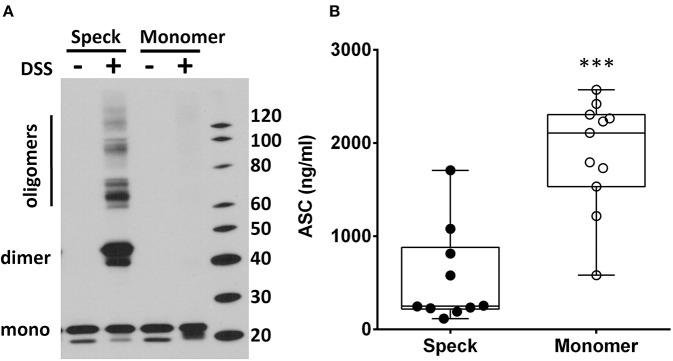
ASC speck formation diminishes ASC detection by ELISA. THP-1 cell lysate (3 × 10^8^cells/ml) was incubated either at 37°C (for speck generation) or 4°C (monomer maintenance) (from *n* = 7 experiments) and then left alone or cross-linked with DSS. ASC concentration and oligomerization status was analyzed by immunoblot **(A)** and ELISA **(B)** where detection was statistically suppressed in specks compared to monomers, ***p < 0.0001.

Inflammasome activation generates oligomeric ASC specks dependent upon PYD and CARD domain mediated polymer formation (27–29). Therefore, we evaluated whether the ASC ELISA can accurately detect the oligomeric forms of ASC. To test this, we compared ASC monomers to ASC oligomers [prepared from THP-1 cells as previously outlined (21)]. Briefly, THP-1 cells generate oligomers of ASC as confirmed by crosslinking with DSS when lysed at 3 × 10^8^/ml on ice and then warmed. In contrast, THP-1 cells lysed at the same concentration, but kept on ice throughout, do not generate oligomers (Figure 4A). Assaying these samples for ASC by ELISA at equal cell lysate concentrations demonstrated a significant reduction in detectability of ASC oligomers compared to monomer samples [251 [124–880] vs. 2,109 [710–2,306] ng/ml, median with interquartile range, *p* < 0.0001] (Figure 4B). This result suggests that ASC's antigenic epitopes are hidden in tightly packed ASC specks or that large ASC complexes are washed away during ELISA washing steps. However, as noted in Figure 1B, BALF does not show a discrepancy between the ELISA and immunoblot quantitation of ASC. Thus, the relative inability of the ELISA to detect ASC specks compared to immunoblots (Figure 4) and the lack of this discrepancy in BALF measurements (Figure 1) imply that BALF ASC is largely void of oligomeric ASC—the traditional trademark of inflammasome activation.

### Chemical Crosslinking Does Not Detect BALF Oligomers of ASC

**Figure 5:**
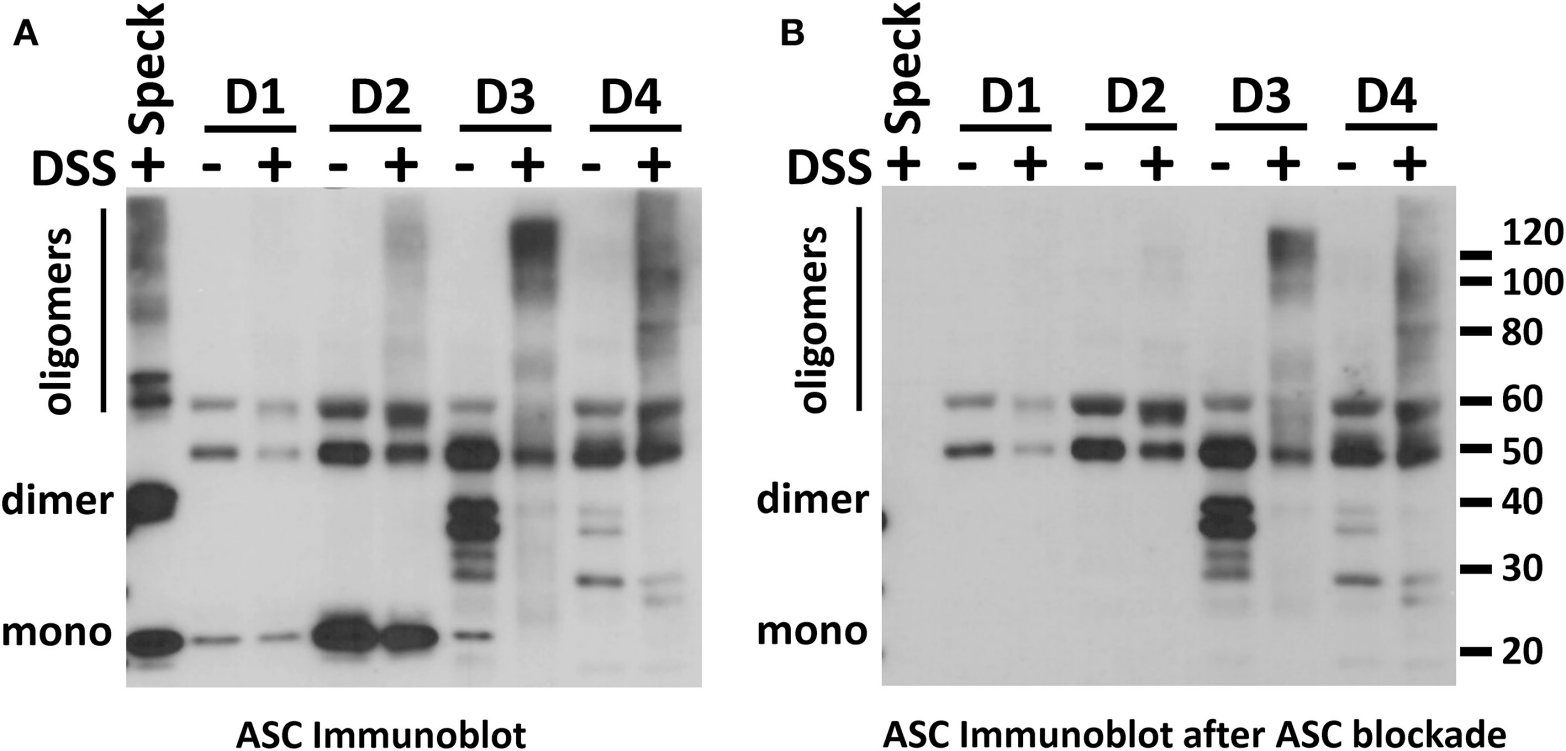
No ASC oligomers are detected in normal human BALF. BALFs from four donors were evaluated for the presence of ASC oligomers using DSS crosslinking by ASC immunoblot **(A)**. A positive control for ASC dimerization and oligomerization was specks induced by *in vitro* ASC speck assembly as previously described (21). To control for non-specific signals, a parallel immunoblot as in **(A)** was stained with rabbit polyclonal antibody that had been precleared of anti-ASC activity by excess recombinant ASC **(B)**. A blink comparator animation showing this effect is in the Supplemental Material.

To further confirm the lack of ASC specks (oligomers), in BALF we used immunoblots combined with chemical crosslinking to characterize the conformational status of the BALF ASC. Although monomers of BALF ASC were clearly visible, no oligomers of ASC were observed after crosslinking with DSS (Figure 5A). We did detect high molecular weight signals in some BALF samples which has been previously reported (11). However, these signals were not the classical dimer and trimer molecular weights for ASC. More critically, pre-blocking the ASC antiserum with recombinant ASC did not clear the high molecular weight signals present in the BALF but did eliminate detection of all monomer, dimer and trimeric forms from positive control specks as expected (Figure 5B, Supplementary Video 1 with blink comparator animation). This finding supports our conclusion that the high molecular weight signals in our BALF samples are non-specific. Thus, most if not all BALF ASC is present in the monomeric, non-speck form.

### Centrifugation Concentrates *in vitro* Generated ASC Specks but Does Not Concentrate BALF ASC

**Figure 6:**
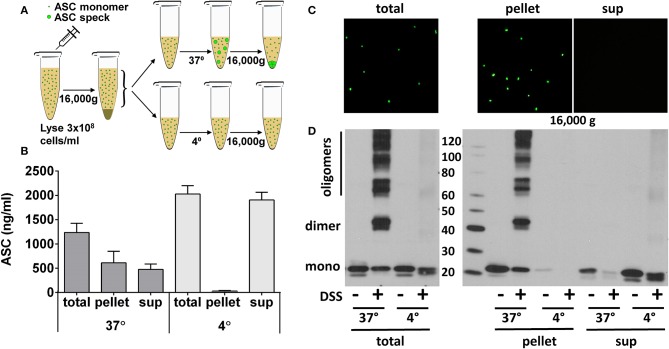
ASC specks are effectively pelleted by high-speed centrifugation. ASC oligomerization was induced in cell extract of THP-1 cells stably expressing YFP-ASC or endogenous ASC. ASC specks, generated as in Figure 4, were further pelleted by centrifugation at 16,000 g for 10 min to allow comparison of ASC detection in resulting pellet or supernatant as in schema **(A)**. Samples were measured by ASC ELISA in wild-type ASC expressing THP-1 cells **(B)**, observed under fluorescent microscopy for YFP-ASC expressing cells **(C)**, or cross-linked and immunoblotted for ASC in wild-type THP-1 cells **(D)**.

Since ASC specks pellet at high speed centrifugation, we next subjected BALF and freshly generated YFP-ASC monomers and specks to centrifugation at 16,000 g (Figure 6A, outlines procedure). YFP-ASC specks were completely pelleted when visualized by fluorescent microscopy (Figure 6C). To test that this observation is not an artifact of the YFP tag, we also generated ASC specks from wild type THP-1 cells analyzed them by ELISA (Figure 6B) and by immunoblots (Figure 6D). As expected, the ASC oligomers were relegated to the pelleted fraction whereas the monomeric ASC remained in the supernatant. Thus, centrifugation can be used to differentiate ASC specks from monomeric cell free ASC.

Having established centrifugation as a reliable means to separate specks, we used BALF aliquots available from our HIV cohort. Centrifuging BALF at speeds that pull down ASC specks did not concentrate ASC as measured by immunoblot nor concentrate a DSS cross-linkable form of ASC in the pelleted fraction (Supplementary Figure 2). These findings further confirm the absence of ASC specks in cell free BALF.

### ASC From BALF in Patients With Pneumonia

**Figure 7:**
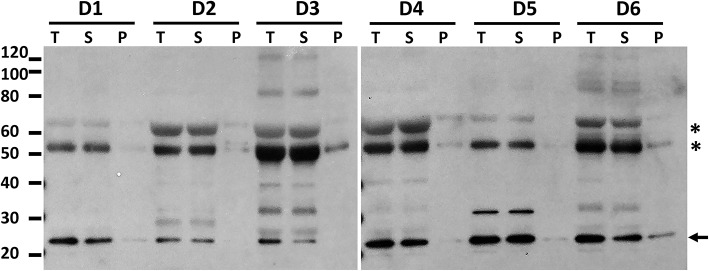
High speed centrifugation and protein crosslinking shows that BALF ASC in pneumonia subjects is the soluble monomeric form. Hospitalized patients undergoing diagnostic bronchoscopy for presumed pneumonia (cohort 3) consented to have residual BALF studied for ASC. Shown are 6 random donors whose fluid was spun at 500 g to remove cells and then the cell free fluid (T, total) was spun at 16,000 g for 10 min to separate pelleted material (P) from its supernatant, i.e., soluble material (S). Cell free fluid (T), supernatant of high speed spin (S), and pellet after spin (P) were subjected to crosslinking with DSS and then assessed for ASC by immunoblot. Asterisks represent non-specific bands as previously characterized in Figure 5 and arrow denotes the monomeric ASC band.

Finally, we investigated whether patients undergoing BAL for suspected pneumonia (cohort 3) showed evidence of ASC speck formation as these subjects had the most elevated levels of ASC (Figure 2). Having demonstrated that high speed centrifugation is a reliable method for concentrating ASC specks, we again utilized centrifugation of BALF to concentrate ASC oligomers from six subjects. No ASC oligomers were detected in BALF from these patients with suspected pneumonia (Figure 7). Although 9 of the 40 pneumonia suspects had detectable BALF IL-1β by ELISA, there was no correlation between ASC and the levels of IL-1β in BALF (*p* = 0.42) and we found no oligomers after crosslinking and immunoblots in 3 samples selected for having the highest ELISA detectable IL-β (data not shown). These findings suggest that, even in subjects with active lung inflammation, the monomeric form of ASC is the predominant form of ASC in cell-free lung lining fluid.

## Discussion

Both monomeric and oligomeric forms of extracellular ASC have been linked to mononuclear phagocytes and inflammation in *in vitro* and *in vivo* models (10, 11, 13, 14, 30). Although the presence of cell-free ASC has been presumed to represent prior inflammasome activation events, supportive studies predominantly used models in which macrophages had been induced to undergo pyroptosis by a TLR priming event followed by NLRP3 activation via ATP or live bacteria (10, 11, 13, 14). However, whether ASC is released from lung macrophages in the normal course of lung surveillance and whether ASC is released as monomers independent of oligomeric forms has not been extensively studied. Since the lung airspace represents a uniquely accessible *in vivo* site for the study of macrophage and ASC relationships, we set out to determine the quantity and characteristics of extracellular ASC recovered from the human lung airway in health and disease.

First of all, we were surprised to discover that the levels of ASC in normal healthy lungs are dramatically elevated. In comparison to normal plasma, lung lining fluid ASC levels in normal, healthy, non-smokers are about 100 times higher when corrected for the dilution from the BAL procedure (22). These high levels of extracellular ASC in lung lining fluid were unexpected.

Since functional ASC has been attributed to the oligomeric, speck form of ASC (11, 14), we needed to determine if our ELISA could detect oligomeric ASC. To do this, we generated ASC specks from wild type and YFP-ASC expressing THP-1 cells and then confirmed with fluorescent microscopy and immunoblots that ASC specks are sedimented by centrifugation at 16,000 g. However, we found that BALF ASC measurements were not changed by centrifugal speck removal. Because we discovered that our ELISA is unable to accurately detect ASC specks, we turned to immunoblot quantitation which we found able to detect both monomeric and oligomeric ASC (Figure 4). Nevertheless, subjecting BALF samples to concentration by sedimentation did not enrich ASC detection by immunoblotting, consistent with monomeric BALF ASC.

Another approach we took to determine if BALF ASC contained polymeric ASC was chemical crosslinking—a process that stabilizes oligomers for detection by immunoblots. Again, both stored and freshly obtained samples lacked the classical dimer, trimer and oligomeric forms after crosslinking. We conclude that the predominant form of ASC in human BALF is the monomeric form and thus not likely part of an inflammasome-triggered ASC speck.

Having characterized the form of the ASC as monomeric and confirming elevated levels in lung fluid, we looked for evidence that ASC might have physiologic significance. Thus, we took advantage of our HIV smoker cohort that had undergone detailed pulmonary function testing to ask if ASC levels might relate to lung function impairment. We found that ASC levels were directly related to the concentration of macrophages recovered, and to salivary cotinines, a measure of active smoking. Congruent with these relationships BALF ASC levels negatively associated with lung diffusion capacity (which is an index of functioning alveolar capillary units). This finding is relevant since smoking and macrophage numbers are clearly associated with the development of chronic obstructive lung disease where loss of diffusion capacity is typical (31–33).

Lastly, we turned to hospitalized patients undergoing diagnostic BALs for presumed lung infections since there was a greater chance for inflammasome activation in the acutely ill. Here we found significantly elevated levels of ASC in the BALF of these patients compared to our healthy HIV+ and HIV- cohorts. However, it is noteworthy that again we found no evidence of speck formation by crosslinking studies.

There are noteworthy differences between our lung results and that of other reports that need comment pertaining to ASC specks in lung (11) or blood (13, 30). These reports provide evidence for the presence of ASC specks in lung and blood. But unlike our quantitative focus using standardized enzyme linked immunoassays for measurement and chemical crosslinking to confirm speck oligomers, these reports relied largely upon the use of flow cytometry. Like Franklin we found lung ASC detection to correlate with evidence of COPD, but we did not find discrete evidence of cell free ASC specks in BALF as they reported. Like Baroja-Maze (13) we believe that specks are largely if not exclusively cell bound (10, 17, 34). This distinction is critical to understanding the function of ASC specks because cell-speck associations contain a host of potentially pro-inflammatory mediators that may function independently from the speck itself.

Technical details can largely explain the differences between these reports. Flow cytometry methods, though highly sensitive, may suffer from non-specificity which may be hard to differentiate. Like the prior report (11) we found high molecular weight forms in BALF after cross-linking. However, the high molecular weight immunoblot signals from our BALF samples were not blocked by recombinant ASC while purified ASC specks were blocked. Additionally, our BALF samples were frozen only after cell removal to avoid freeze thawing of ASC rich macrophages. These technical details were not discussed in the prior report (11). Of course, separating cells from BALF before storage might remove extracellular ASC specks. However, this low speed centrifugation does not bring down cell free ASC specks as we noted in our THP-1 cell generated YFP-ASC specks. Thus, we do not believe that cell free ASC specks were lost during cell separation and that sample handling may indeed explain our discrepancies.

Another way specks might be lost is that they are actually tightly associated with pyroptotic cell remains. Pyroptotic macrophages may be short-lived and rapidly cleared by efferocytosis along with their associated specks. Lastly, it may be that ASC specks are unstable and readily dissolve into monomeric forms in the milieu of the lung micro-environment.

Nevertheless, the high monomeric airspace ASC levels maintained against a negative plasma gradient remain worthy of further study because they suggest a physiological role for the released monomeric ASC. For example, it is conceivable that monomeric ASC may function distinctly from filamentous polymeric ASC. Interactions of caspase-1 with monomeric ASC may not induce caspase-1 activation. That is, monomeric ASC interactions may not provide the caspase-1 dimerization and molecular proximity that ASC polymer platforms use to spark inflammasome activation (9). In that scenario one could envision that excess extracellular monomeric ASC might isolate caspase-1 in an inhibited conformation.

Although we suspect that alveolar macrophages are the source of the excess ASC in BALF, based upon the close correlation demonstrated in Figure 3A, we cannot exclude the possibility that lung epithelial cell ASC may be an important source. One could hypothesize that chronic macrophage abundance in the lung of smokers causes injury to lung epithelial cells leading to the release of ASC. Such a hypothesis is intriguing as it provides a link between BALF ASC levels and the loss of lung diffusion capacity we noted in our smoking HIV cohort (Figure 3B).

In summary, our findings are the first to accurately quantitate ASC in lung lining fluid which demonstrated remarkably elevated concentrations of ASC in BALF compared to plasma. The high BALF ASC concentrations suggest a steady accumulation relative to clearance in the air space. In contrast to the prior report (11), ASC specks were not found in BALF of healthy non-smoking and smoking subjects nor in patients with suspected pneumonia. However, in agreement with that report we did find a clear relationship between inflammatory conditions and the level of ASC detected. Finally, the association of ASC with loss of lung diffusing capacity in smokers suggests a possible direct effect of the macrophage derived monomeric ASC on lung physiology. Thus, future studies that seek to understand the functional significance of the lung extracellular ASC are warranted.

## Data Availability Statement

The datasets generated for this study are available on request from the corresponding authors.

## Ethics Statement

The studies involving human participants were reviewed and approved by The Ohio State University Institutional Review Board - IRB 2005H0197, IRB 2015C008, IRB2016H0009. The patients/participants provided their written informed consent to participate in this study.

## Author Contributions

MG, CM, and MW conceived and designed experiments, analyzed the data, and wrote the paper. EP, MT, CS, M-AS, SM, AS, PS, and PD collected samples, interpreted data, wrote, edited, and reviewed the manuscript. CM, EP, and MG performed the experiments.

### Conflict of Interest

The authors declare that the research was conducted in the absence of any commercial or financial relationships that could be construed as a potential conflict of interest.
