# Supplementary material for: Inflammasome Adaptor ASC Is Highly Elevated in Lung Over Plasma and Relates to Inflammation and Lung Diffusion in the Absence of Speck Formation
Source: Front Immunol. 2020 Mar 19;11:461. doi: 10.3389/fimmu.2020.00461 (PMC7096349; doi:10.3389/fimmu.2020.00461)
Supplement: Supplementary file 1 [file Data_Sheet_1.PDF]

Supplementary Table I.

Comparisons of HIV Smoker Parameters (Cohort 2) with Lung Function Measures*

	Pulmonary Function							
	**DLCO**		**FEV1**		**TLC**		**RV**	
BAL parameters	p	r	p	r	p	r	p	r
ASC (ng/ml)	**0.0038**	**-0.34**	0.42	-0.096	0.59	0.064	0.29	0.12
Protein (μg/ml)	0.96	0.005	0.59	0.065	0.91	-0.01	0.99	-.0.002
Macs/ml	**0.05**	**-0.23**	0.60	-0.063	0.49	-0.084	0.44	-0.092
Lymphocytes %	0.52	0.077	**0.032**	**0.25**	0.65	-.054	0.95	0.008
Neutrophils %	0.18	-0.16	**0.04**	**-0.24**	0.78	-.034	0.61	-0.060
Smoking parameters								
Cotinine (ng/ml)	**0.017**	**-0.29**	0.055	-0.23	0.48	0.086	**0.042**	**0.24**
Pack-yr	0.45	0.096	0.07	-0.23	0.66	0.056	0.32	0.13
Subject parameters								
Age, yr.	0.94	0.008	0.053	-0.23	0.25	-0.14	0.58	-0.066
BMI, kg/m^2	0.18	0.16	0.23	0.14	0.81	-0.029	0.26	0.13

*Shown are the p value (bolded if $p \leq 0.05$) and Pearson correlation coefficients for BAL, smoking, and subject parameters versus pulmonary function measures (analyzed as percent predicted for lung diffusion capacity (DLCO), forced expiratory volume in 1 second (FEV1), total lung capacity (TLC) and residual volume (RV) for the HIV smoker cohort, n = 74. Cotinine is from saliva, macrophage cell numbers/ml of BALF (Macs/ml), and lymphocyte and neutrophil percent is of the total cells recovered.
