# Supplementary material for: Inflammasome Adaptor ASC Is Highly Elevated in Lung Over Plasma and Relates to Inflammation and Lung Diffusion in the Absence of Speck Formation
Source: Front Immunol. 2020 Mar 19;11:461. doi: 10.3389/fimmu.2020.00461 (PMC7096349; doi:10.3389/fimmu.2020.00461)
Supplement: Supplementary file 2 [file Data_Sheet_2.PDF]

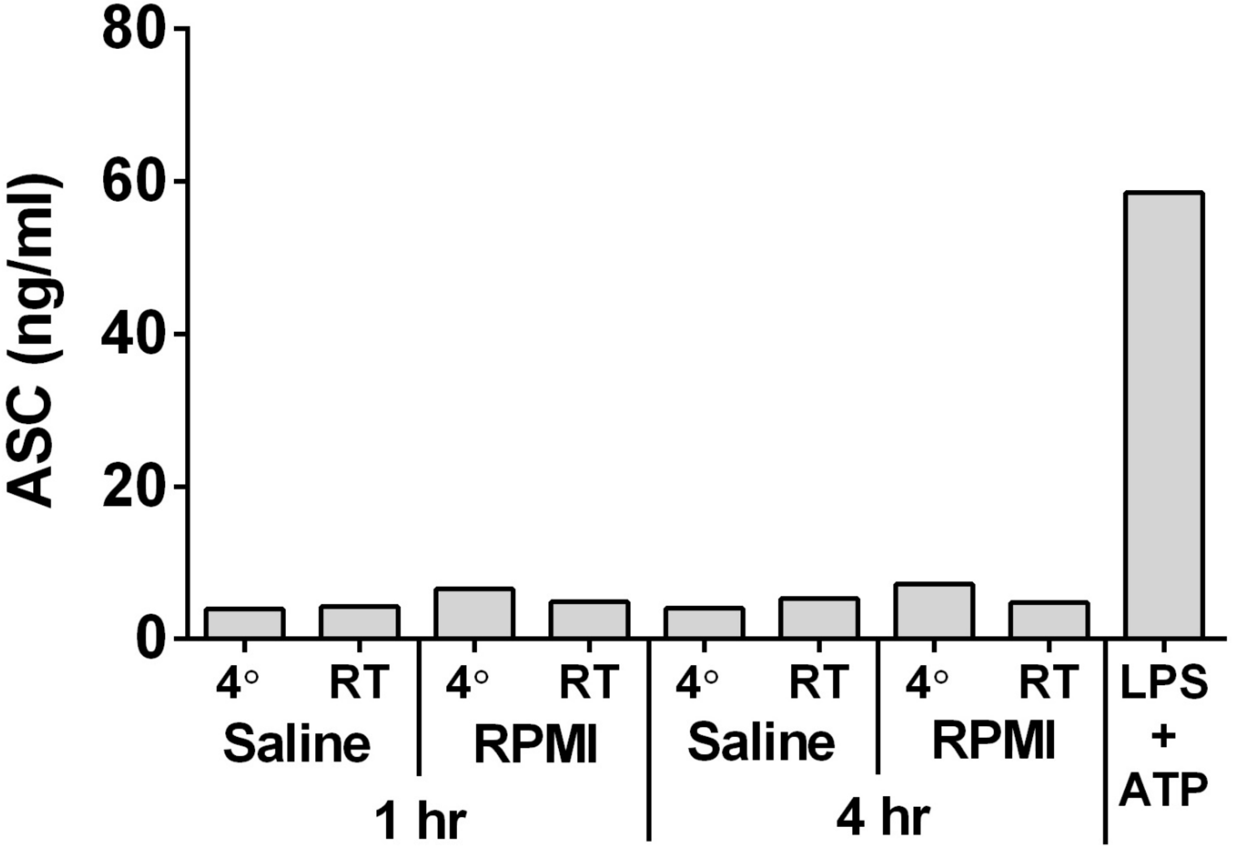

Suppl. Fig. 1 Mononuclear cell ASC release not related to incubation in saline at room temperature. THP-1 cells were washed and placed in culture with either serum free RPMI or saline for 1 and 4 h at 106 cells/ml at 4°C or room temperature (RT). Supernatants were harvested and subjected to ASC ELISA. As positive control same cells were activated with LPS 1 µg/ml for 30 min followed by ATP 5mM for additional 30 min and harvested samples used in same ELISA.
