# Supplementary material for: Inflammasome Adaptor ASC Is Highly Elevated in Lung Over Plasma and Relates to Inflammation and Lung Diffusion in the Absence of Speck Formation
Source: Front Immunol. 2020 Mar 19;11:461. doi: 10.3389/fimmu.2020.00461 (PMC7096349; doi:10.3389/fimmu.2020.00461)
Supplement: Supplementary file 3 [file Data_Sheet_3.PDF]

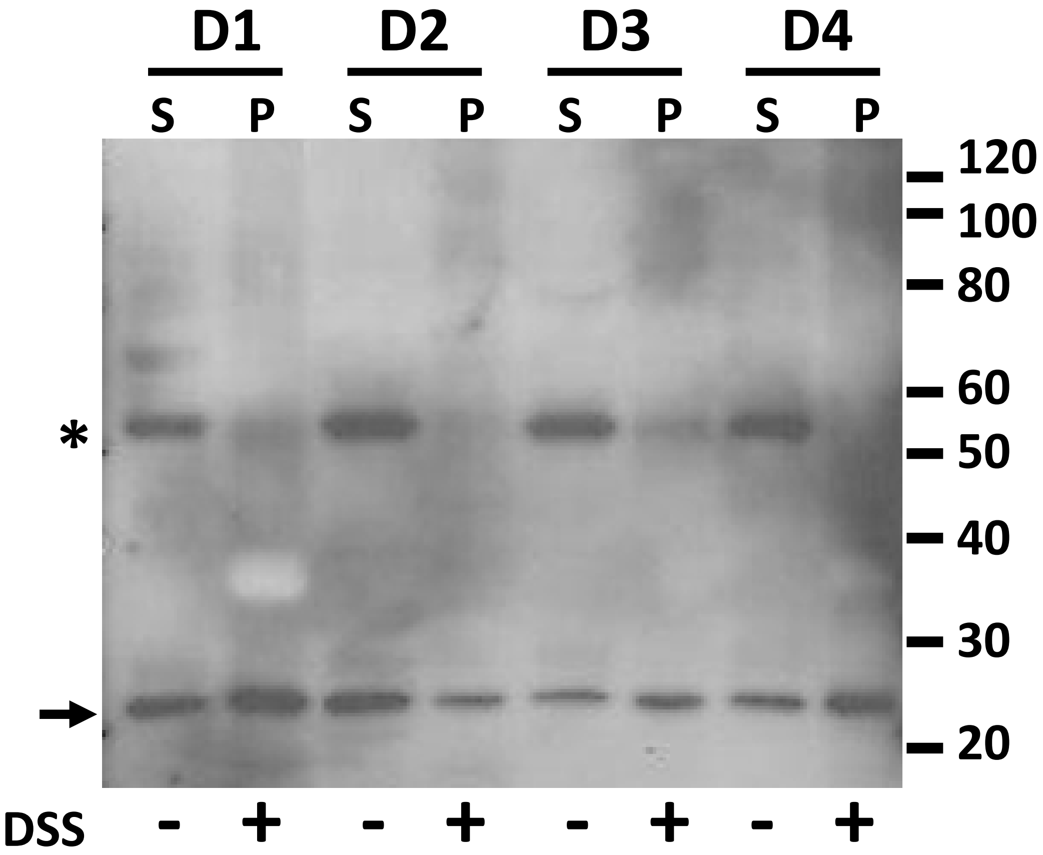

Suppl. Fig. 2 Pelleting of BALF does not concentrate polymeric ASC forms. BALF aliquots (5 ml) with ELISA detectable ASC from 4 healthy HIV donors were centrifuged at 16,000 g for 10 min. The supernatant and the concentrated pellet (which was treated with DSS to document polymer formation) are shown.
