# Supplementary material for: Inflammasome Adaptor ASC Is Highly Elevated in Lung Over Plasma and Relates to Inflammation and Lung Diffusion in the Absence of Speck Formation
Source: Front Immunol. 2020 Mar 19;11:461. doi: 10.3389/fimmu.2020.00461 (PMC7096349; doi:10.3389/fimmu.2020.00461)
Supplement: Supplementary file 4 [file Data_Sheet_4.docx]

**Supplementary Video 1**. **Neutralization of ASC antibody shows nonspecific signal in BALF**. Open file Video 1.MP4 to run blink comparator animation to allow easy comparison between two ASC immunoblots from BAL supernatants from four random donors in which one blot was probed with rabbit ASC antibody alone or the same antibody after pretreatment with recombinant MBP-ASC to neutralize the ASC specific antibody.
